# Targeted Isolation of Coumarins From *Sideritis* Species Based on Antiviral Screening and Untargeted Metabolomics

**DOI:** 10.1002/pca.3531

**Published:** 2025-04-04

**Authors:** Ekaterina‐Michaela Tomou, Olivier Engler, Antonios Chrysargyris, Nikolaos Tzortzakis, Helen Skaltsa, Corinna Urmann

**Affiliations:** ^1^ Section of Pharmacognosy and Chemistry of Natural Products, Department of Pharmacy, School of Health Sciences National and Kapodistrian University of Athens Athens Greece; ^2^ Organic‐Analytical Chemistry Weihenstephan‐Triesdorf University of Applied Sciences Straubing Germany; ^3^ Spiez Laboratory Federal Office for Civil Protection Spiez Switzerland; ^4^ Department of Agricultural Sciences, Biotechnology and Food Science Cyprus University of Technology Limassol Cyprus; ^5^ TUM Campus Straubing for Biotechnology and Sustainability Technical University of Munich Straubing Germany

**Keywords:** antiviral effect, GC–MS, Lamiaceae, metabolite profile, mountain tea

## Abstract

**Introduction:**

The SARS‐CoV‐2 pandemic has revealed a deficiency in antiviral agents. Plants, traditionally used for respiratory infections, are valuable sources of antiviral compounds. Such a plant is the *Sideritis* L. taxa (mountain tea), traditionally used against cold and cough.

**Objectives:**

Accordingly, this study aimed to investigate the potential protective effects of dichloromethane extracts from *Sideritis* species against SARS‐CoV‐2.

**Materials and Methods:**

Eight *Sideritis* extracts were tested in an *in vitro* pretreatment assay to assess the protective effect against SARS‐CoV‐2. Therefore, infectious virus particles were pre‐incubated with the extract, then incubated with Vero E6 cells to finally measure cell viability as a surrogate for virus infection. Untargeted analyses (GC–MS and LC‐PDA‐HRESIMS) were performed to determine metabolite profiles.

**Results:**

Using an orthogonal approach that combines untargeted metabolomics and biological data from a screening assay, we characterized the phytochemical profiles of the different extracts and prioritized samples for targeted isolation. The dichloromethane extract of *Sideritis cypria* exhibited a notable protective effect. Untargeted analysis revealed coumarins as key compounds, with varying amounts across *Sideritis* species. Accordingly, fractionation of extract resulted in the isolation of two coumarin derivatives. Structure elucidation was performed using one‐ and two‐dimensional nuclear magnetic resonance experiments. The coumarin, more abundant in *S. cypria* , demonstrated a slight protective effect in the SARS‐CoV‐2 pretreatment assay.

**Conclusion:**

This study highlights the antiviral effects of *Sideritis* taxa, although further investigations are necessary to clarify the full potential of the herb. Additionally, the methodology presented herein can serve as a valuable resource for future phytochemical investigations focused on coumarin content within *Sideritis* genus.

## Abstract 2

This study explores the antiviral efficacy of eight *Sideritis* taxa, evaluating the potential protective effects of the dichloromethane extracts against SARS‐CoV‐2 *in vitro*. Combining untargeted metabolomics with biological screening data, the phytochemical profiles of the extracts were analyzed. This approach identified two coumarin derivatives and led to the first‐time isolation of a trimethoxy‐dimethyl‐coumarin from the *Sideritis* genus, highlighting the plants' potential in antiviral applications and contributing to the understanding of the medicinal properties.

## 1 Introduction

In December 2019, a pandemic linked to a virus from the coronavirus family appeared in Wuhan (China). The coronavirus, named SARS‐CoV‐2 (severe acute respiratory syndrome coronavirus 2), was rapidly spread worldwide and shattered healthcare systems around the world [1]. Although the severity of cases is decreasing today, the likelihood of further outbreaks remains, as does the potential for new variants to emerge in the future [2]. In response to the pandemic, the urgent need for effective treatments has spurred researchers to explore a range of innovative approaches.

About 34% of all Food and Drug Administration (FDA)‐approved antiviral therapeutics are either natural products, directly derived from natural products or traceable to naturally occurring chemical compounds. The proportion of natural products represented in antiviral small‐molecule drugs rises to nearly 77%, underscoring the crucial role of the secondary metabolome of organisms in the discovery and development of antiviral therapies [3]. Since plant extracts show antiviral efficacy [4, 5], the use of herbal medicine for therapeutic purposes should not be underestimated. A number of traditional medicines has been used for the treatment of COVID‐19 in a variety of countries as an adjunct to modern medical therapies [6, 7, 8, 9, 10].

The genus *Sideritis* L. (Lamiaceae family) occurs in tropical and temperate regions of the northern hemisphere and mainly in the Mediterranean area [11]. The name “Sideritis” is derived from the Greek word iron (“sideros”); this nomenclature resonates with the historical utilization to treat wounds arising from iron weapons [11]. *Sideritis* spp. are extensively applied in several traditional medicines around the world. As outlined in the monograph of the European Medicines Agency (EMA), the traditional use of *Sideritis* preparations (also known as mountain tea) is associated with the relief of mild gastrointestinal discomfort and coughs related to the common cold [12]. In Balkan countries, *Sideritis scardica* Griseb. has been used mainly for treating lung diseases and cough of different origins (like asthma and bronchitis) [13]. Previous studies on *Sideritis* extracts have documented a multitude of pharmacological activities, including antioxidant, antiinflammatory, neuroprotective, anticholinesterase and cytotoxic effects [11, 14, 15, 16]. However, few studies have investigated the antiviral effects of *Sideritis* extracts and components [17, 18, 19]. Specifically, *Sideritis hirsuta* L. showed an effect on the influenza H3N2 virus [17], while the dichloromethane extract of *Sideritis perfoliata* L. subsp. *perfoliata* demonstrated an activity against the herpes simplex virus (HSV) [18]. Furthermore, the antiviral effects against human parainfluenza virus type 2 (HPIV‐2) of the acetone extract of *Sideritis lycia* Boiss. & Heldr. and of the isolated *ent*‐kaurene diterpenes were evaluated [19]. Phytochemical studies on the genus *Sideritis* reported a broad range of chemical components such as terpenoids, iridoids, coumarins, flavonoids, phenylethanoid glycosides, and others [11, 20, 21, 22, 23]. Considering the documented antiviral efficacy, an investigative study was conducted to ascertain the potential protective effects of dichloromethane extracts derived from *Sideritis* species against SARS‐CoV‐2 infection.

## 2 Material and Methods

### 2.1 Plant Material

The aerial parts of *Sideritis* taxa were collected during the flowering stage in June and July in 2018, 2019, and 2021. A comprehensive list of the *Sideritis* samples is provided in Table 1. Following harvesting, the samples were air‐dried and stored under nondestructive conditions. *S. sipylea*, *S. raeseri* subsp. *attica*, *S. clandestina* subsp. *clandestina*, and *S. raeseri* subsp. *raeseri* were authenticated by Prof. Th. Constantinidis and Dr. K. Goula and voucher specimens (Lytra & Skaltsa 01–04, respectively) were deposited in the Herbarium of the Department of Pharmacognosy and Chemistry of Natural Products, School of Pharmacy, NKUA. *S. euboea* and *S. scardica* were provided by ELGO Dimitra and authenticated by Dr. P. Chatzopoulou (codes 19–17 and GRC017, respectively). *S*. *cypria* and *S. perfoliata* subsp. *perfoliata* were produced from genetic material originated from the mother plantations of the National Department of Agriculture at Athalassa (Cyprus). The comminuted air‐dried aerial parts of each *Sideritis* taxa were extracted with dichloromethane at room temperature, concentrated to dryness and stored at a temperature of 4°C in the dark until further use.

**TABLE 1:** List of the investigated Sideritis taxa in different collection dates, locations, and abbreviations used.

*Sideritis* taxa	Country	Location	Collection date/Origin	Abb.
*S. euboea* Heldr.	Greece	Hellenic Agriculture Organisation “Demeter”‐ Thessaloniki	2021–07/cultivated	SC1
*S. scardica* Griseb.	Greece	Hellenic Agriculture Organisation “Demeter”‐ Thessaloniki	2021–07/cultivated	SC2
*S. raeseri* Boiss. et Heldr subsp. *attica* (Heldr.) Pap. Et Kok	Greece	Mount Parnitha‐ Attiki	2021–07/cultivated	SC3
*S. cypria* Post.	Cyprus	Athalassa‐ Nicosia	2019–06/cultivated	SC4
*S. perfoliata* L. subsp. *perfoliata*	Cyprus	Athalassa‐ Nicosia	2018–06/cultivated	SC5
*S. raeseri* Boiss. & Heldr. subsp. *raeseri*	Greece	Mount Makrikampos‐ Pogoni	2021–07/wild	SW1
*S. clandestina* (Bory & Chaub.) Hayek subsp. *clandestina*	Greece	Mount Taygetus‐ Peloponnese	2021–06/wild	SW2
*S. sipylea* Boiss.	Greece	Mount Kerkis‐ Samos	2019–06/ wild	SW3

For isolation, comminuted air‐dried plant material of *S. euboea* (*m* = 197.8 g) was extracted at room temperature with dichloromethane and concentrated to dryness to yield a residue of *m* = 2.2 g.

### 2.2 Isolation of Compounds

A part of the dichloromethane extract of *S. euboea* (*m* = 0.5 g each run, in total 2.0 g) was loaded on silica gel (1:10, *m* = 5.0 g) (VWR, Darmstadt, Germany) and was prefractionated by flash column chromatography, using as eluent mixtures of increasing polarity (hexane:ethyl acetate) to yield finally 12 fractions (F1–F12). A Puriflash 4250 system (Interchim, Montlucon, France) was used with a volume of 75 mL for each solvent composition and a flow of 15 mL/min. The fraction (*m* = 287 mg), which contained the coumarins, was subjected to solid phase extraction (SPE) in order to filtrate as a pre‐step for further isolation. C_18_ cartridges of 1 g were used. After activation of the stationary phase with methanol, water and methanol, the sample was diluted in acetonitrile and was then loaded and washed gradually by five cartridge volumes with acetonitrile. Afterwards, the obtained sample was evaporated to dryness under reduced pressure and the residue (*m* = 190.41 mg) was taken to HPLC for isolation and afforded 10 fractions. The fractions were collected manually according to the signal at *λ* = 320 nm, and the different runs were combined to yield fractions 1 (t_R_ 14.15 min; *m* = 3.2 mg) and 2 (t_R_ 14.63 min; *m* = 0.6 mg). A HPLC‐system (Shimadzu, Duisburg, Germany) with a column oven equipped with a C_18_ column (Knauer, Berlin, Germany) Eurosphere‐C_18_, 100 μm 8 × 250 mm was used with a gradient of solvents A (water+0.1% formic acid) and B (acetonitrile+0.1% formic acid). 8‐methoxycoumarsabin (compound **1**) was obtained from AnalytiCon Discovery.

### 2.3 GC–MS

Analysis was performed using a Shimadzu QP2010SE, operating in the EI mode (70 eV) equipped with a split injector and a fused silica Optima 5HT capillary column (30 m × 0.25 mm I. D., film thickness: 0.25 μm). Temperature was increased from 50 to 340°C at a rate of 10°C/min. Helium was used as a carrier gas at a flow rate of 1.05 mL/min on the column, with a split ratio of 20. The injector temperature was set to 300°C, the interface temperature to 340°C, and the ion source temperature to 250°C. The MS was used in scan mode from m/z 50–850. Silylation was performed employing BSTFA reagent (Macherey‐Nagel). Of each sample, 8 mg was mixed with 320 μL of dry pyridine and 80 μL of BSTFA in screw cap glass tubes and were placed in a silica oil bath at *T* = 70°C for 75 min. Each derivatized sample was cooled to room temperature for 30 min and centrifuged for 10 min at 15000 rpm. The supernatant was directly analyzed using GC–MS. The identification of the compounds was based on comparison of the mass spectra with those of the NIST14 database.

### 2.4 LC‐PDA‐HRESIMS

Analysis was performed similar to Tomou *et al.* (2023) [21]. Briefly, a Shimadzu system with two LC‐20 ad pumps, an autosampler, column oven, and PDA detector was used. A reversed‐phase Phenomenex Kinetex C_18_ column (2.1 × 100 mm, 2.6 μm) was employed with solvents A (water + 0.1% formic acid) and B (acetonitrile + 0.1% formic acid) at a 0.4 mL/min flow rate and 30 C. A gradient from 15% to 95% B over 35 min was applied. HRESIMS was recorded in negative ionization mode (−3.0 kV) on an IT TOF (Shimadzu), with MS^1^ (m/z 100–1000) and MS^2^ (m/z 50–1000) at 10 and 30 ms of ion accumulation times, respectively. CID energy was set to 50%.

### 2.5 NMR

NMR spectra were recorded in CDCl_3_ on Bruker AvanceCore (400 MHz for ^1^H‐NMR and 100 MHz for ^13^C‐NMR) and Jeol JNM‐ECS‐400 (400 MHz for ^1^H‐NMR and 100 MHz for ^13^C‐NMR) spectrometers. Chemical shifts are expressed in δ (ppm) and were referenced to the solvent signal at δ_H_ 7.26 ppm and δ_C_ 77.2 ppm for ^1^H‐NMR and ^13^C‐NMR, respectively. NOESY (Nuclear Overhauser Effect Spectroscopy), HSQC (Heteronuclear Single Quantum Coherence), and HMBC (Heteronuclear Multiple Bond Coherence) experiments were performed using standard Bruker or Jeol microprograms.

### 2.6 Antiviral Assay

The antiviral assay was performed as reported in Vahekeni *et al.* (2024) [6]. Briefly, plant extracts were resuspended in DMSO to a concentration of 25 mg/mL and diluted to 200, 66.7, 22.2, 7.4, and 2.5 μg/mL in cell culture medium (2%‐FCS‐MEM). The plant extracts, V = 50 μL of each concentration, were distributed in duplicates into upper half of a flat bottom 96 well plate (TPP, Trasadigen, Switzerland), to assess antiviral activity, and the same concentrations of plant extracts were distributed into the lower half of the 96 well plate, to assess toxicity of extracts. On each plate, infected but untreated cells were included as virus controls and untreated and uninfected cells as cell control. Further controls included serial dilutions of Remdesivir and DMSO as a diluent control. Plates were transferred to the BSL‐3 laboratory and 100 PFU SARS‐CoV‐2 (2019‐nCoV/IDF0372/2020) in a volume of 50 μL of culture medium was added to upper half of the plate as well as to the wells foreseen for the virus control (VC), while to the lower half of the 96 well plate and to the cell control, a volume of 50 μL of culture medium was added. Plates were incubated for 1 h at 37°C and 5% CO_2_, and a volume of 100 μL of Vero E6 cell suspension (2 × 10^5^ cells/mL in 2%‐FCS‐MEM) were added to each well. Plates were incubated for 72 h at 37 °C and 5% CO_2_ and cell viability was determined by CellTiter‐Glo® Luminescent Cell Viability Assay (Promega) according to the manufacturers protocol. Luminescence was measured using the GloMax instrument (Promega).

### 2.7 Software

The software package OriginPro 2021b (64‐bit) SR2 was employed for the purposes of data analysis and graph preparation. The protective effect and cell viability were calculated as follows:

$$\text{protective effect}=\frac{{\text{luminescence}}_{\text{compound treated cells}}-{\text{luminescence}}_{\text{virus treated cells}}}{{\text{luminescence}}_{\text{untreated}\;\mathrm{ce}l\mathrm{ls}}-{\text{luminescence}}_{\text{virus treated cells}}}$$

$$\text{cell viability}=\frac{{\text{luminescence}}_{\text{compound treated cells}}}{{\text{luminescence}}_{\text{untreated cells}}}$$

The feature tables were generated via the GNPS web interface [24]. A straightforward Python 3 script was utilized to narrow down the features based on the maximum presence in the *S. cypria* extract and the maximum abundance in the feature table (.csv file).

## 3 Results and Discussion

A preliminary antiviral screening was initiated on dichloromethane extracts derived from eight different *Sideritis* taxa (Table 1) in an *in vitro* pretreatment assay designed to assess the protective effect against SARS‐CoV‐2. The virus particle was preincubated with the extract, and following further incubation with Vero E6 cells, the viability of the cells was determined using CellTiterGlo®. To ascertain the impact of the extracts on cellular viability, the effect on cells treated solely with the extracts was assessed in parallel.

**FIGURE 1:**
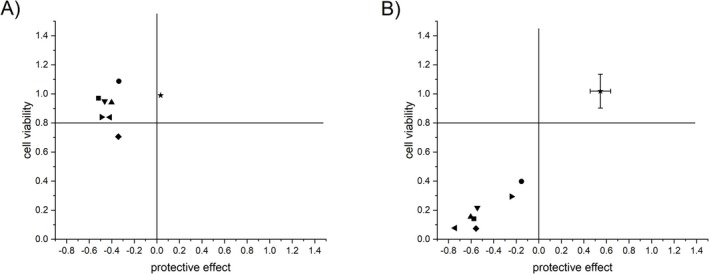
Effect on cell viability and protective effect against SARS‐CoV‐2 in the *in vitro* pretreatment assay of DCM extracts of different *Sideritis* taxa in a concentration of (A) 11.1 μg/mL and (B) 33.3 μg/mL (■SC1, ◀SC2, ▶SC3, ◆SC5, ▲SW1, ▼SW2, ●SW3; *n* = 1),(*n* = 3 for ★SC4 ( *S. cypria* )).

*Sideritis* dichloromethane (DCM) extracts, redissolved in DMSO, were tested in five different concentrations (1.2–100 μg/mL) to ensure that at least one concentration of *Sideritis* extract had no impact on cell viability and that the protective effect could be determined. The results indicated that the DCM extracts exhibited a notable reduction in cell viability of Vero E6 cells, reaching a level of 2%–5% of that observed in untreated cells at the concentration of 100 μg/mL (data not shown). At a concentration of 33.3 μg/mL, the DCM extract of *S. cypria* demonstrated no effect on cell viability and a protective effect of 57% ± 9% in the *in vitro* pretreatment assay concerning SARS‐CoV‐2 infection (Figure 1). In addition, all extracts revealed no effect on cell viability and no protective effect, with the exception of *S. cypria* at a concentration of 11.1 μg/mL (Figure 1). Previous studies have investigated the cytotoxic effect of diverse *Sideritis* taxa extracts on a range of different cell lines [11, 14, 25, 26, 27], indicating the potential involvement of flavonoids [25] and *ent*‐kaurene diterpenes [19, 28, 29] as possible active components.

**FIGURE 2:**
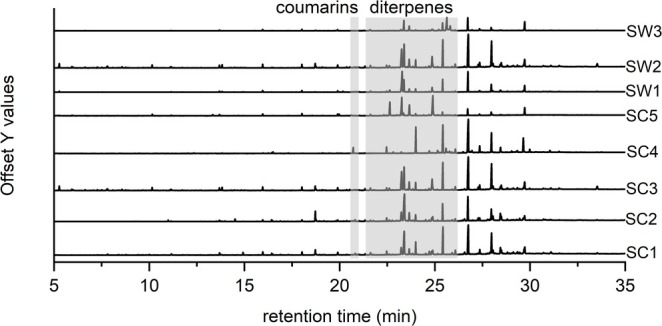
Stacked GC–MS chromatograms of dichloromethane extracts of different *Sideritis* taxa.

Based on the preliminary screening results (Figure 1), the extracts were further investigated using untargeted metabolomics, including gas chromatography–mass spectrometry (GC–MS) and liquid chromatography‐photodiode array‐high‐resolution electrospray ionization mass spectrometry (LC‐PDA‐HRESIMS) with the objective of identifying similarity and differences of metabolite profiles. As a first step, the dichloromethane extracts were analyzed by GC–MS, which revealed notable differences in the chemical compositions (Figure 2). The components were classified into the following categories: fatty acids, alkanes/alkenes, diterpenes, triterpenoids, and phytosterols [20].

**FIGURE 3:**
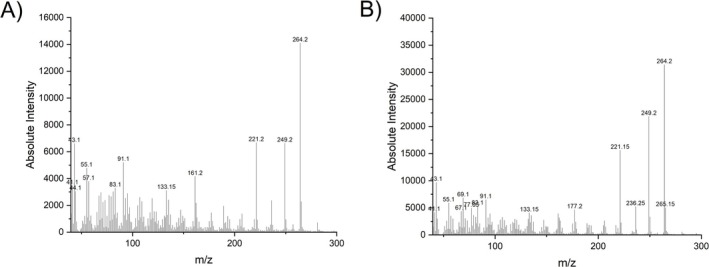
GC–MS spectra of (A) compound **1** and (B) compound **2**.

**SCHEME 1:**
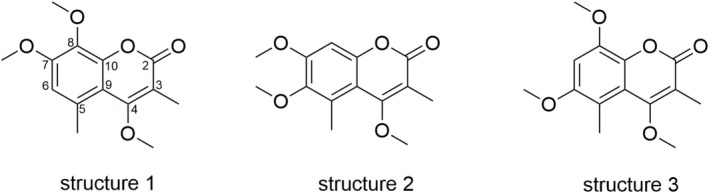
Hypothesized possible structures of the two coumarins.

The GNPS [24] derived feature table of GC–MS data (Figure 2) from investigated *Sideritis* samples was filtered to retain only those features exhibiting the highest abundance in the dichloromethane extract of *S. cypria* . Further filtering (abundance > 10^5^) resulted in the identification of 32 features, representing 3% of the original 1092 features. These features could be assigned to alkanes, phytosterols, and coumarins. Using the Wiley12/NIST20 library, the signal at the retention time of 20.71 min was identified as a coumarin (compound **1**). Furthermore, the feature table generated from GC–MS analysis of derivatized *Sideritis* samples included 977 features, which could be limited to 24 (abundance>10 [6]) and assigned to alkanes, coumarins, fatty acids, and phytosterols. The original mass and retention time were observed concerning compound **1**, despite the derivatization procedure to obtain the trimethylsilylethers of possible hydroxyl groups. This led to the assumption that no hydroxyl group is present in the structure of compound **1**. Subsequently, an LC‐PDA‐HRESIMS analysis was performed and the obtained data, mainly a λ_max_ of 317 nm and a m/z of 265.1057 (C_14_H_16_O_5_ [M + H]^+^ m/z_predicted_ 265.1071), supported the hypothesis of the presence of a coumarin derivative. A comparison of the mass and the proposed sum formula with that of unsubstituted coumarin reveals a substitution pattern comprising three methoxy groups and two methyl groups. Further investigation of the other extracts using a XIC (m/z 264.05) in GC–MS and a XIC (m/z 265.1) in LC‐HRESIMS indicated the presence of a second coumarin (compound **2**). The similarity of the fragmentation patterns of both compounds in GC–MS (Figure 3) led to the assumption of three possible structures (Scheme 1).

Structure **1**, known as 8‐methoxycoumarsabin, has been previously identified in the leaves of *Juniperus sabina* [30] and the roots of *Leucas inflata* [31], *Clutia lanceolata* [32], and *Sideritis pullulans* [28]. Structure **2** has been reported on *Clutia lanceolata* [32] and *Clutia abyssinica* [33], while no literature data were found for structure **3**.

**FIGURE 4:**
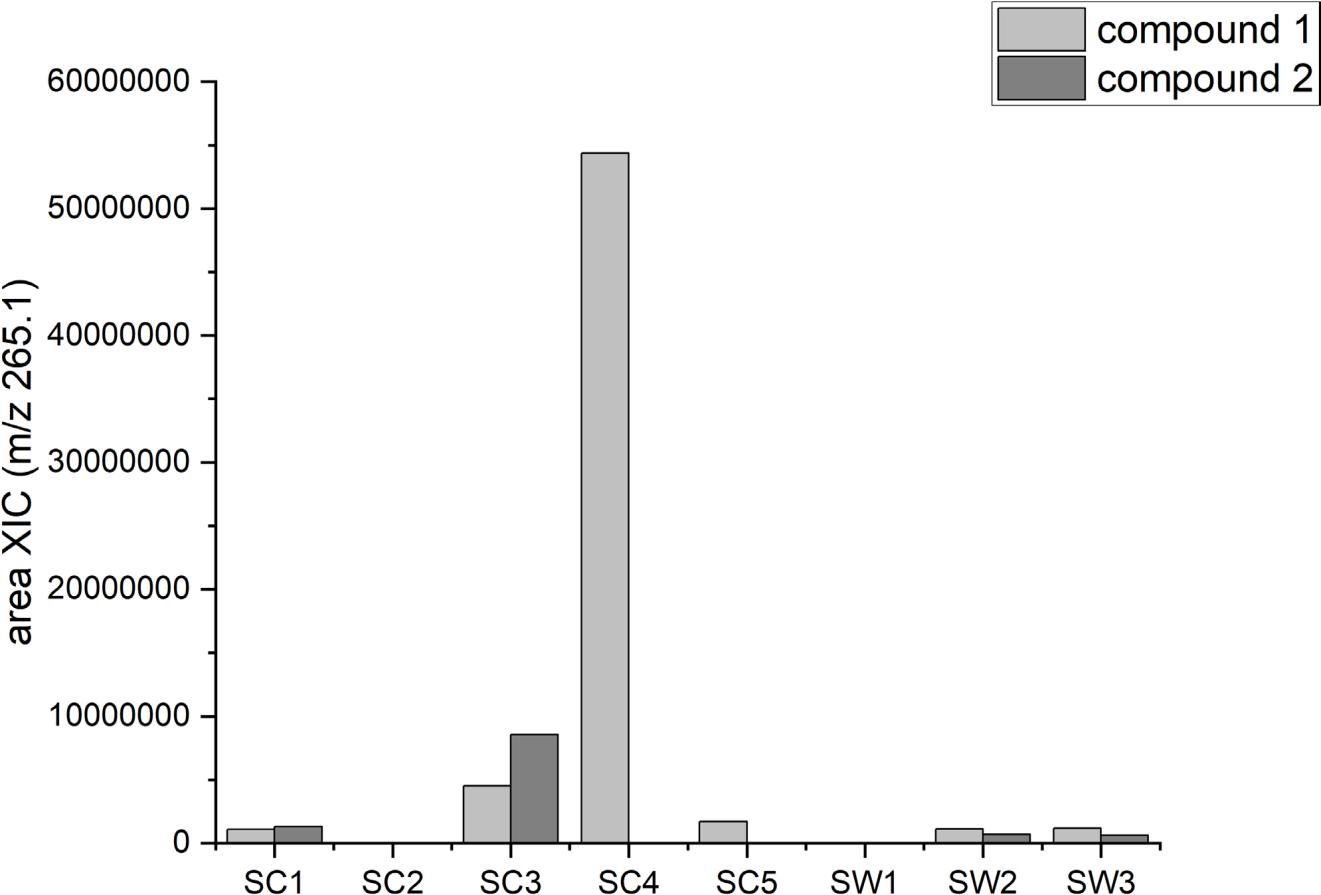
Areas of compounds **1** and **2** from LC/MS, using XIC m/z 265.1.

To compare the amounts of the two coumarins (Figure 4) in the dichloromethane extracts derived from divers *Sideritis* taxa, LC–MS and the areas of the XIC m/z 265.1 were used. Both coumarin isomers (compounds **1** and **2**) were found in *S. euboea* (SC1), *S. raeseri* subsp. *attica* (SC3), *S. clandestina* subsp. *clandestina* (SW2), and *S. sipylea* (SW3) (Figure S1). Specifically, the lowest quantities were observed in *S. scardica* (SC2) and *S. raeseri* subsp. *raeseri* (SW1), while only compound **1** was detected in *S. cypria* (SC4) and *S. perfoliata* subsp. *perfoliata* (SC5). It is noteworthy that compound **1** was detected in the dichloromethane extract of *S. cypria* (SC4) at an amount at least 10 times higher than observed in *S. euboea* (SC1). Moreover, it is noteworthy that the coumarins were present in both the dichloromethane extracts of wild‐collected (SW2 & SW3) and cultivated specimens (SC1, SC3, SC4, and SC5) of the *Sideritis* taxa, harvested from diverse geographical locations (Table 1).

**FIGURE 5:**
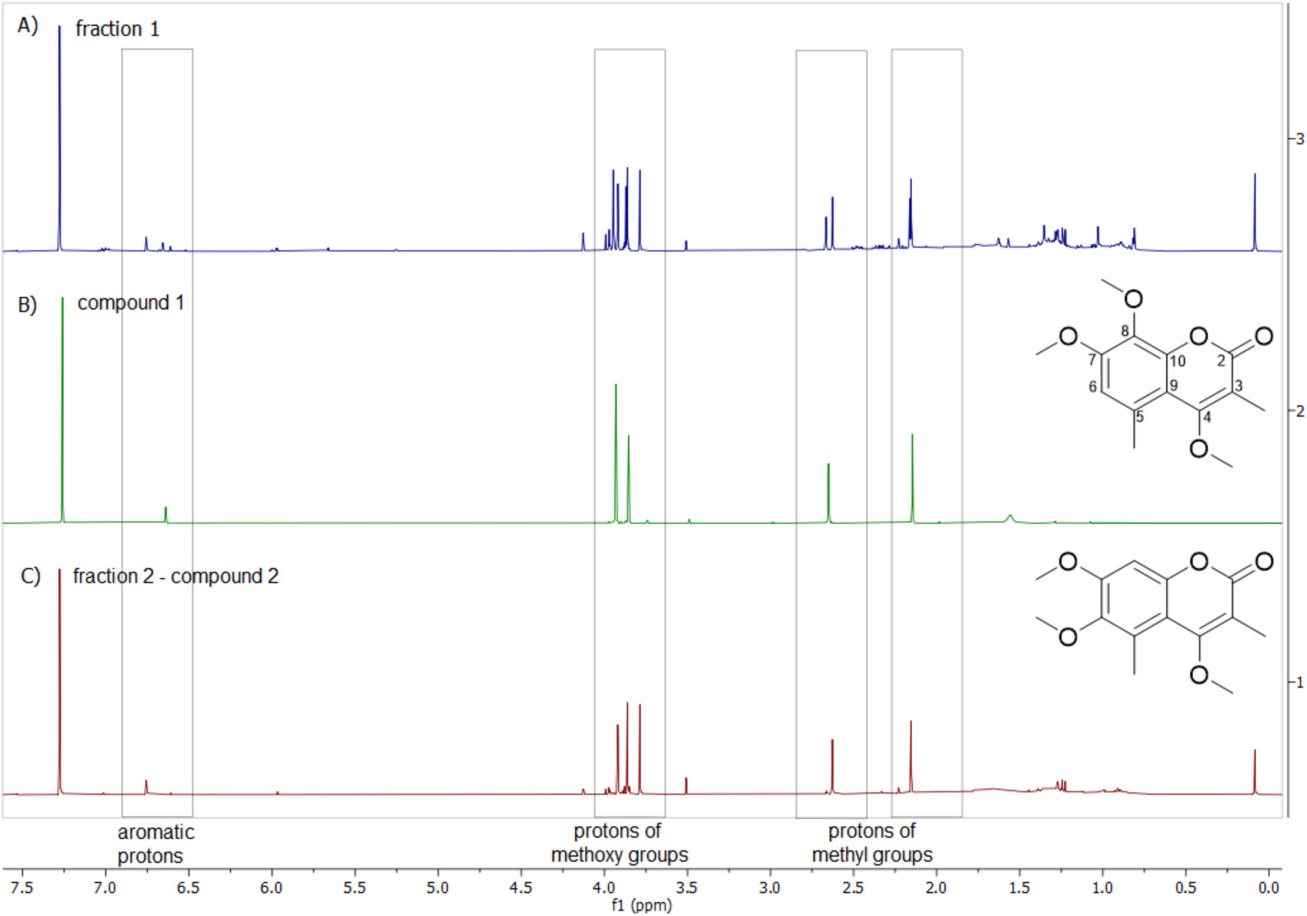
Stacked ^1^H‐NMR spectra of (A) fraction 1, (B) compound **1**, and (C) fraction 2‐compound **2** in CDCl_3_.

In continuation of our previous studies on *S. euboea* [20, 21, 29, 34, 35], the dichloromethane extract of this species was chosen to fractionate and isolate the coumarin isomers. The whole fractionation process was continuously monitored by LC–MS, which enables the characterization of the obtained fractions and any derived subfraction in detail, giving evidence for compounds of interest belonging to coumarins. This approach resulted in two fractions (fractions 1 and 2) from preparative HPLC containing the coumarins in different abundances. These fractions were analyzed using 1D‐ and 2D‐NMR experiments (Figure 5 and Table 2).

**TABLE 2:** ^1^H‐NMR and ^13^C‐NMR spectroscopic data of compounds **1** and **2**; δ_C_/δ_H_ in ppm; δ_H_ (multiplicity) in CDCl_3_.

1			2	
Position	δ_H_	δ_C_	δ_H_	δ_C_
1	—	—	—	—
2	—	164.0	—	164.0
3	—	110.8	—	110.6
3‐CH_3_	2.15 (s)	10.4	2.14 (s)	10.4
4	—	166.7	—	166.8
4‐OCH_3_	3.86 (s)	60.6	3.85 (s)	60.3
5	—	130.8	—	128.4
5‐CH_3_	2.66 (s)	22.0	2.61 (s)	12.5
6	6.63 (br s)	111.7	—	144.3
7	—	153.8	—	155.1
7‐OCH_3_	3.93 (s)	55.9	3.90 (s)	56.0
8	—	134.7	6.74 (br s)	98.2
9	—	110.7	—	109.6
10	—	146.9	—	150.9
6‐OCH_3_	—	—	3.77 (s)	60.3
8‐OCH_3_	3.93 (s)	61.2	—	—

In the ^1^H‐NMR spectrum of fraction 1 (Figure 5A), signals from coumarins were detected, including (i) aromatic protons (δ_H_ range of 6.74–6.55), (ii) protons of methoxy groups (δ_H_ range of 4.0–3.7), and (iii) protons from aromatic methyl groups (δ_H_ range of 2.66–2.12). In addition, carbon signals were observed at δ_C_ range of 112.8–98.2 (corresponding to aromatic protons), 62.0–55.9 (assigned to methoxy groups), and 22.0–10.4 (corresponding to aromatic methyl groups) in the HSQC spectrum.

In order to identify the two coumarins, the compound with structure 1 (known as 8‐methoxycoumarsabin) was obtained from AnalytiCon Discovery. The retention time and fragmentation pattern in both GC–MS and LC–MS were found to be similar to those of compound **1** in *S. cypria* . In an effort to interpret the different signals of the coumarins observed, the NMR spectra of compound **1** (Figures 5B and S2–S6) were recorded and compared with existing literature data [30, 36]. In the ^1^H‐NMR spectrum, one aromatic proton at δ_H_ 6.63 (H‐6), two aromatic methyl groups at δ_H_ 2.15 (3‐CH_3_) and δ_H_ 2.66 (5‐CH_3_), and three methoxy groups at δ_H_ 3.86 (4‐OCH_3_) and δ_H_ 3.93 (7‐OCH_3_/8‐OCH_3_) were detected. Furthermore, the HSQC spectrum showed correlations at δ_H_ 6.63 (br s, H‐6)/ δ_C_ 111.7 (C‐6), δ_H_ 3.86 (s, 3H, 4‐OCH_3_)/ δ_C_ 60.6 (4‐OCH_3_), δ_H_ 3.93 (br s, 6H)/δ_C_ 55.9 (7‐OCH_3_), 61.2 (8‐OCH_3_), δ_H_ 2.15 (s, 3‐CH_3_)/ δ_C_ 10.4 (3‐CH_3_), and δ_H_ 2.66 (s, 5‐CH_3_)/δ_C_ 22.0 (5‐CH_3_). The NOESY spectrum confirmed the proximity of the aromatic proton at δ_H_ 6.63 (H‐6) to the 7‐OCH_3_ (δ_H_ 3.93) and the 5‐CH_3_ (δ_H_ 2.66). Moreover, NOESY correlations were identified between the 4‐OCH_3_ (δ_H_ 3.86)/3‐CH_3_ (δ_H_ 2.15), as well as between the 4‐OCH_3_ (δ_H_ 3.86)/5‐CH_3_ (δ_H_ 2.66); the latter was observed to be weaker. In the HMBC spectrum, the aromatic proton at 6.63 (H‐6) demonstrated correlations with quaternary carbon signals at δ_C_ 110.7 (C‐9) and δ_H_ 134.7 (C‐8). In addition, the HMBC spectrum showed correlations from methyl protons at position 3 (δ_H_ 2.15) to carbons at δ_C_ 110.8 (C‐3), 164.0 (C‐2), and 166.7 (C‐4), indicating the presence of α‐pyrone moiety typical of a substituted coumarin [28]. In the HMBC spectrum, the protons of the methoxy groups at δ_H_ 3.93 showed correlations with two quaternary carbons at δ_C_ 153.8 (C‐7) and 134.7 (C‐8), while the protons of 4‐OCH_3_ demonstrated correlations to carbon at δ_C_ 166.7 (C‐4). By meticulously comparing the NMR spectra of fraction 1 with those of the purchased coumarin, the initial hypothesis regarding the presence of coumarins in fraction 1 was confirmed. Therefore, compound **1**, namely, 8‐methoxycoumarsabin, was identified in fraction 1. Although this constituent was previously reported in the roots of *Sideritis pullulans* [28], it is mentioned for the first time in aerial parts of *S. euboea* (SC1), *S. raeseri* subsp. *attica* (SC3), *S. clandestina* subsp. *clandestina* (SW2), *S. sipylea* (SW3), *S. cypria* (SC4), and *S. perfoliata* subsp. *perfoliata* (SC5).

Afterwards, the NMR spectra of fraction 2 (Figures 5C and S7–S11) were obtained, revealing the presence of the other coumarin isomer (compound **2**). The ^1^H‐NMR data of compound **2** (Table 2) showed the presence of typical signals of coumarin derivative with one aromatic proton at δ_H_ 6.74 (H‐8), two aromatic methyls at δ_H_ 2.14 (3‐CH_3_) and 2.61 (5‐CH_3_), and three methoxy groups at δ_H_ 3.90 (7‐OCH_3_), 3.85 (4‐OCH_3_), and 3.77 (6‐OCH_3_), giving correlations with carbons signals in the HSQC spectrum at δ_C_ 98.2, 10.4, 12.5, 56.0, 60.3, and 60.3, respectively. From the NOESY spectrum, the aromatic proton at δ_H_ 6.74 (H‐8) was confirmed to be close to the 7‐methoxy group at δ_H_ 3.90. Furthermore, NOESY correlations were also identified among 3‐CH_3_ (δ_H_ 2.14) /4‐OCH_3_ (δ_H_ 3.85), 4‐OCH_3_ (δ_H_ 3.85)/5‐CH_3_ (δ_H_ 2.61), and 5‐CH_3_ (δ_H_ 2.61)/6‐OCH_3_ (δ_H_ 3.77). In the HMBC spectrum, the aromatic proton at 6.74 (H‐8) displayed correlations with four quaternary carbon signals at δ_C_ 109.6 (C‐9), 144.3 (C‐6), 150.9 (C‐10), and 155.1 (C‐7). Furthermore, the HMBC spectrum showed correlations from methyl protons at position 3 (δ_H_ 2.14) to carbons at δ_C_ 110.6 (C‐3), 164.0 (C‐2), and 166.8 (C‐4), indicating the presence of α‐pyrone moiety typical of a substituted coumarin [28]. According to the above results, the structure of compound **2** was elucidated as 4,6,7‐trimethoxy‐3,5‐dimethylcoumarin [33]. This coumarin derivative was found in *Clutia lanceolata* [32] and *C. abyssinica* [33], but this is the first report on *Sideritis* genus.

**FIGURE 6:**
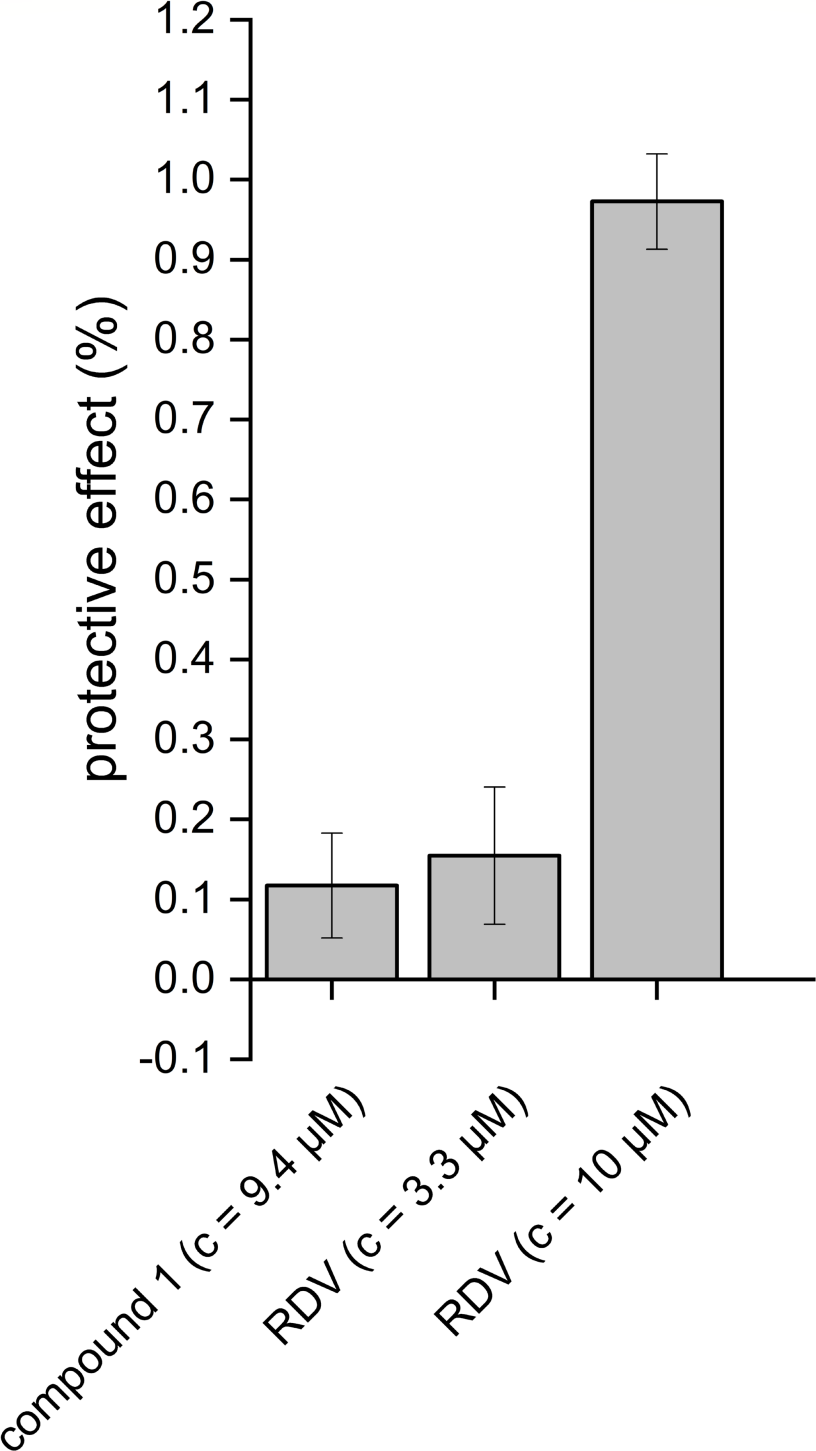
Screening of the antiviral effect of compound **1** and the positive control Remdesivir (RVD) in the in vitro virus pretreatment assay (compound 1 *n* = 2; RDV *n* = 3).

Finally, the protective effect of compound **1**, which was found to be the main coumarin in *S. cypria* , was determined in the pretreatment assay against SARS‐CoV‐2 infection (Figure 6). The results of the virus pretreatment assay indicate that 8‐methoxycoumarsabin (compound **1**) exhibits a modest degree of protective effect at a concentration of 2.5 μg/mL (*c* = 9.4 μM), which is comparable to the protective effect observed for Remdesivir (positive control) at a concentration of 3.3 μM. Further investigation is required to determine whether the protective effect of the dichloromethane extract of *S. cypria* could be exclusively attributable to the coumarin content. Moreover, the antiviral activity of these coumarins remains to be fully explored, as compound **1** was only identified as a potential protective agent in an *in vitro* screening assay.

In general, coumarins exhibit various pharmacological activities, including antioxidant, antiinflammatory, anticoagulant, antiviral antimicrobial, neuroprotective, antidiabetic, anticonvulsant, and antiproliferative [37, 38, 39]. Furthermore, coumarins appear to be active *in vitro* against several viruses, including human immunodeficiency virus, influenza virus, and hepatitis virus [40]. The different substituents and conjugates in the structure of coumarins could change the efficacy against viruses [39]. Especially methoxylated coumarins such as scoparone are already documented to demonstrate antiviral effects, for instance, against the hemorrhagic septicemia virus (40% cytopathic effect at a concentration of 100 μg/mL) [41].

Diterpenes, of which different skeleton types are found in the genus *Sideritis* [23], could not be identified as protective components in the bioactivity‐linked analysis in this study. This is in contrast to a study on *Sideritis lycia* in which the antiviral effect of an acetone extract and the isolated diterpenes such as linearol, sidol and isosidol were reported [42]. This discrepancy could be attributed to the fact that the detection of active components in antiviral tests is largely dependent on the specific assay employed [43].

The combination of untargeted metabolomic analysis with antiviral screening facilitated the prioritization of *Sideritis* samples with intriguing phytochemical profiles and potential protective effects against SARS‐CoV‐2 infection in vitro. This approach resulted in the targeted identification of two trimethoxy‐dimethyl‐coumarins with one (compound **2**) being isolated for the first time from the *Sideritis* genus. To date, very few studies have reported the presence of coumarins in *Sideritis* species. Therefore, the present study may facilitate future phytochemical investigations focusing on coumarin content within this genus, benefiting from the methodology reported here. Considering the diverse pharmacological activities of coumarins, these compounds may contribute to the beneficial effects of *Sideritis* species. Additionally, the findings also highlight the antiviral efficacy of *Sideritis* taxa, underscoring the necessity for additional investigation into the potential as antiviral agents.

## Supporting information


**Data S1.** Supporting Information Captions.

**Figure S1.** Stacked LC–MS chromatograms of dichloromethane extracts of different *Sideritis* taxa with positive ionization.
**Figure S2.** ^1^H‐NMR spectrum of compound **1** (CDCl_3_).
**Figure S3.** ^13^C‐NMR spectrum of compound **1** (CDCl_3_).
**Figure S4.** NOESY spectrum of compound **1** (CDCl_3_).
**Figure S5.** HSQC spectrum of compound **1** (CDCl_3_).
**Figure S6.** HMBC spectrum of compound **1** (CDCl_3_).
**Figure S7.** ^1^H‐NMR spectrum of compound **2** (CDCl_3_).
**Figure S8.** ^13^C‐NMR spectrum of compound **2** (CDCl_3_).
**Figure S9.** NOESY spectrum of compound **2** (CDCl_3_).
**Figure S10.** HSQC spectrum of compound **2** (CDCl_3_).
**Figure S11.** HMBC spectrum of compound **2** (CDCl_3_).

## Data Availability

The data that support the findings of this study are available in the supporting information of this article or from the corresponding author upon reasonable request.
