# Supplementary material for: Targeted Isolation of Coumarins From Sideritis Species Based on Antiviral Screening and Untargeted Metabolomics
Source: Phytochem Anal. 2025 Apr 4;36(5):1570–9. doi: 10.1002/pca.3531 (PMC12212021; doi:10.1002/pca.3531)
Supplement: Supplementary file 1 — Data S1. Supporting Information Captions. [file PCA-36-1570-s002.docx]

**Supporting information:** Figure S 1. Stacked LC-MS chromatograms of dichloromethane extracts of different *Sideritis* taxa with positive ionization. Figure S 2. ^1^H-NMR spectrum of compound 1 (CDCl_3_). Figure S 3. ^13^C-NMR spectrum of compound 1 (CDCl_3_). Figure S 4. NOESY spectrum of compound 1 (CDCl_3_). Figure S 5. HSQC spectrum of compound 1 (CDCl_3_). Figure S 6. HMBC spectrum of compound 1 (CDCl_3_). Figure S 7. ^1^H-NMR spectrum of compound 2 (CDCl_3_). Figure S 8. ^13^C-NMR spectrum of compound 2 (CDCl_3_).

Figure S 9. NOESY spectrum of compound 2 (CDCl_3_). Figure S 10. HSQC spectrum of compound 2 (CDCl_3_). Figure S 11. HMBC spectrum of compound 2 (CDCl_3_).
