# Supplementary material for: Targeted Isolation of Coumarins From Sideritis Species Based on Antiviral Screening and Untargeted Metabolomics
Source: Phytochem Anal. 2025 Apr 4;36(5):1570–9. doi: 10.1002/pca.3531 (PMC12212021; doi:10.1002/pca.3531)
Supplement: Supplementary file 2 — Figure S1. Stacked LC–MS chromatograms of dichloromethane extracts of different Sideritis taxa with positive ionization. Figure S2. 1H‐NMR spectrum of compound 1 (CDCl3). Figure S3. 13C‐NMR spectrum of compound 1 (CDCl3). Figure S4. NOESY spectrum of compound 1 (CDCl3). Figure S5. HSQC spectrum of compound 1 (CDCl3). Figure S6. HMBC spectrum of compound 1 (CDCl3). Figure S7. 1H‐NMR spectrum of compound 2 (CDCl3). Figure S8. 13C‐NMR spectrum of compound 2 (CDCl3). Figure S9. NOESY spectrum of compound 2 (CDCl3). Figure S10. HSQC spectrum of compound 2 (CDCl3). Figure S11. HMBC spectrum of compound 2 (CDCl3). [file PCA-36-1570-s001.pdf]

Supplementary Information

Targeted isolation of coumarins from *Sideritis* species based on antiviral screening and untargeted metabolomics

Short title: Coumarins isolation from *Sideritis* species and antiviral screening

Ekaterina-Michaela Tomou[1,2#], Olivier Engler[3], Antonios Chrysargyris[4], Nikolaos Tzortzakis[4], Helen Skaltsa[1], and Corinna Urmann[2,5#*]

[1]Section of Pharmacognosy & Chemistry of Natural Products, Department of Pharmacy, School of Health Sciences, National and Kapodistrian University of Athens, Athens, Greece;
[2]Organic-Analytical Chemistry, Weihenstephan-Triesdorf University of Applied Sciences, Straubing, Germany;
[3]Spiez Laboratory, Federal Office for Civil Protection, 3700 Spiez, Switzerland
[4]Department of Agricultural Sciences, Biotechnology and Food Science, Cyprus University of Technology, Limassol 3036, Cyprus
[5]TUM Campus Straubing for Biotechnology and Sustainability, Technical University of Munich, Straubing, Germany

[#]authors contributed equally
[*]corresponding author: corinna.urmann@hswt.de, corinna.urmann@tum.de

List of contents

Figure S 1. Stacked LC-MS chromatograms of dichloromethane extracts of different Sideritis taxa with positive ionization. 3
Figure S 2. ^{1}H-NMR spectrum of compound **1** ($CDCl_3$). 3
Figure S 3. ^{13}C-NMR spectrum of compound **1** ($CDCl_3$). 4
Figure S 4. NOESY spectrum of compound **1** ($CDCl_3$). 4
Figure S 5. HSQC spectrum of compound **1** ($CDCl_3$). 5
Figure S 6. HMBC spectrum of compound **1** ($CDCl_3$). 5
Figure S 7. ^{1}H-NMR spectrum of compound **2** ($CDCl_3$). 6
Figure S 8. ^{13}C-NMR spectrum of compound **2** ($CDCl_3$). 6
Figure S 9. NOESY spectrum of compound **2** ($CDCl_3$). 7
Figure S 10. HSQC spectrum of compound **2** ($CDCl_3$). 7
Figure S 11. HMBC spectrum of compound **2** ($CDCl_3$). 8

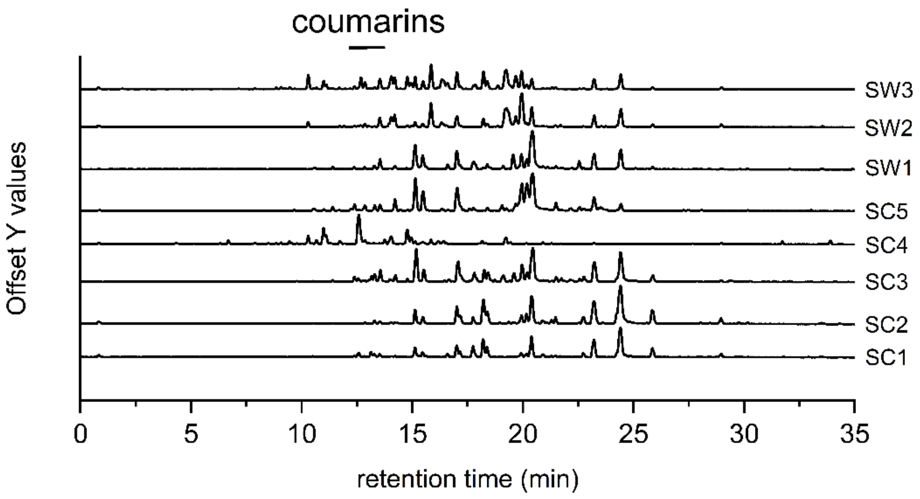

Figure S 1. Stacked LC-MS chromatograms of dichloromethane extracts of different *Sideritis* taxa with positive ionization.

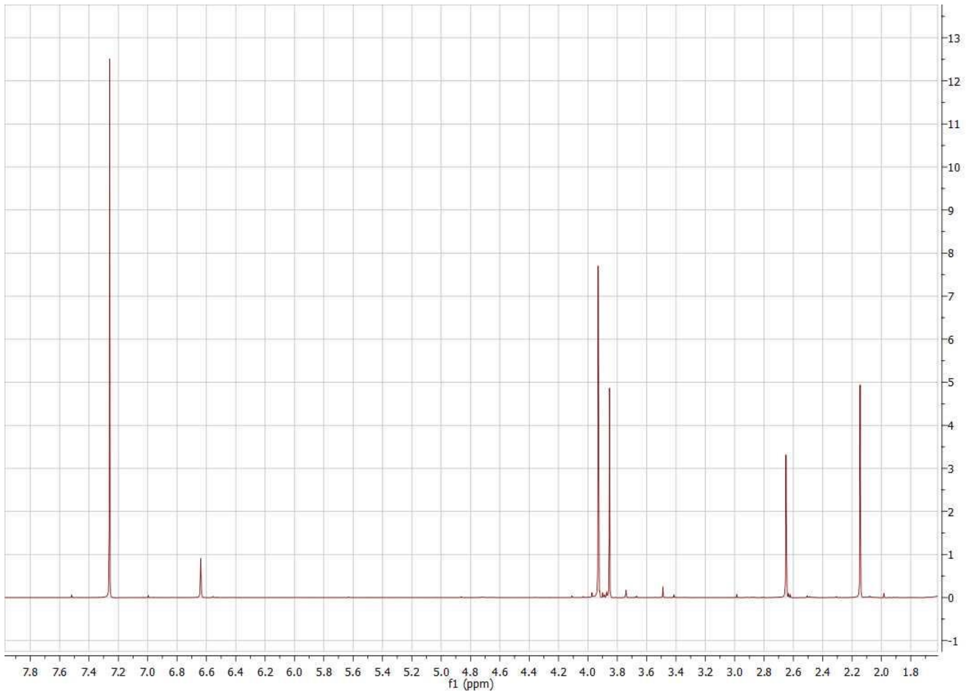

Figure S 2. ^{1}H-NMR spectrum of compound **1** ($CDCl_3$).

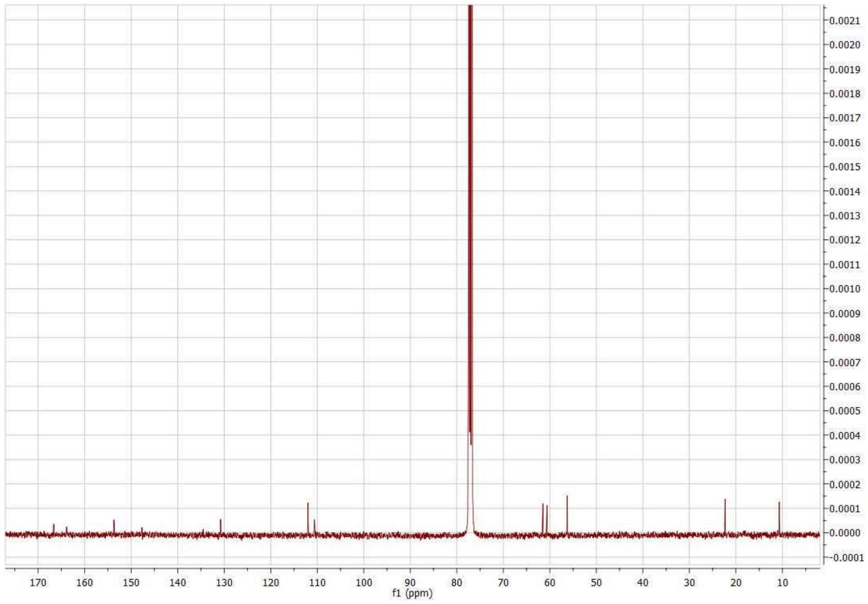

Figure S 3. ^{13}C-NMR spectrum of compound **1** ($CDCl_3$).

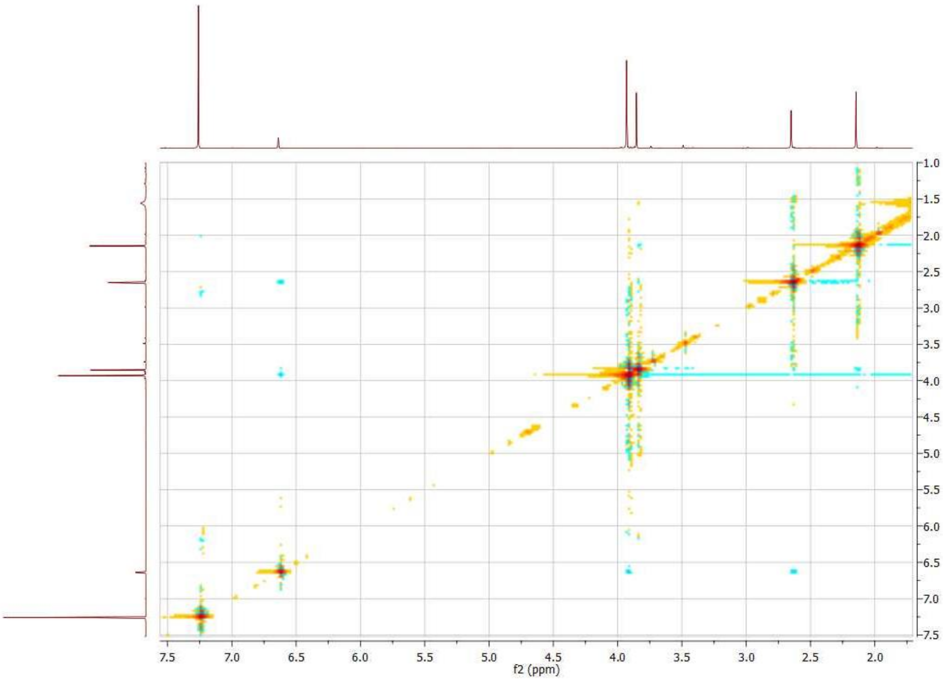

Figure S 4. NOESY spectrum of compound **1** ($CDCl_3$).

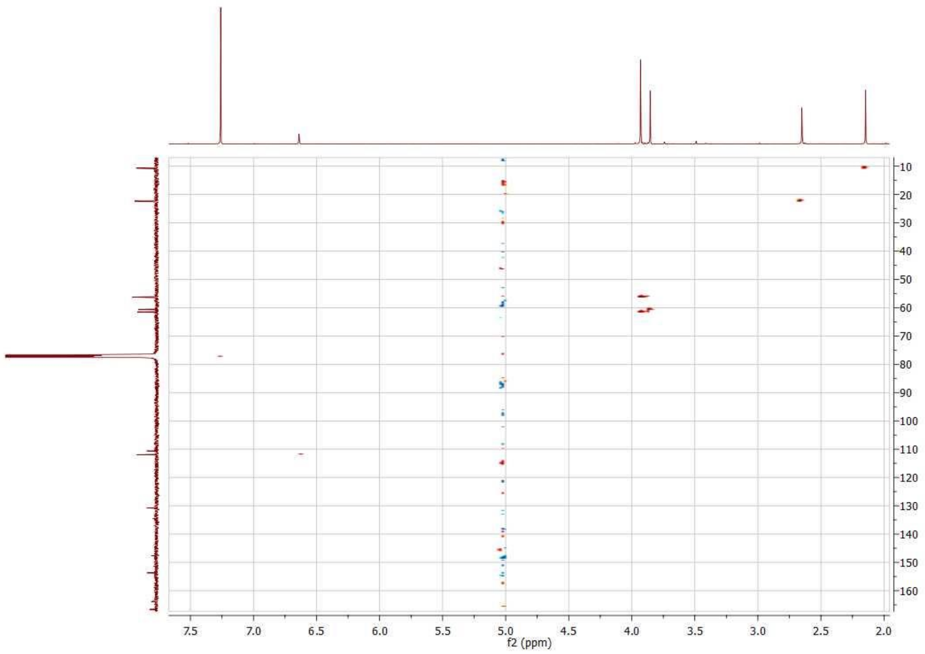

Figure S 5. HSQC spectrum of compound **1** ($CDCl_3$).

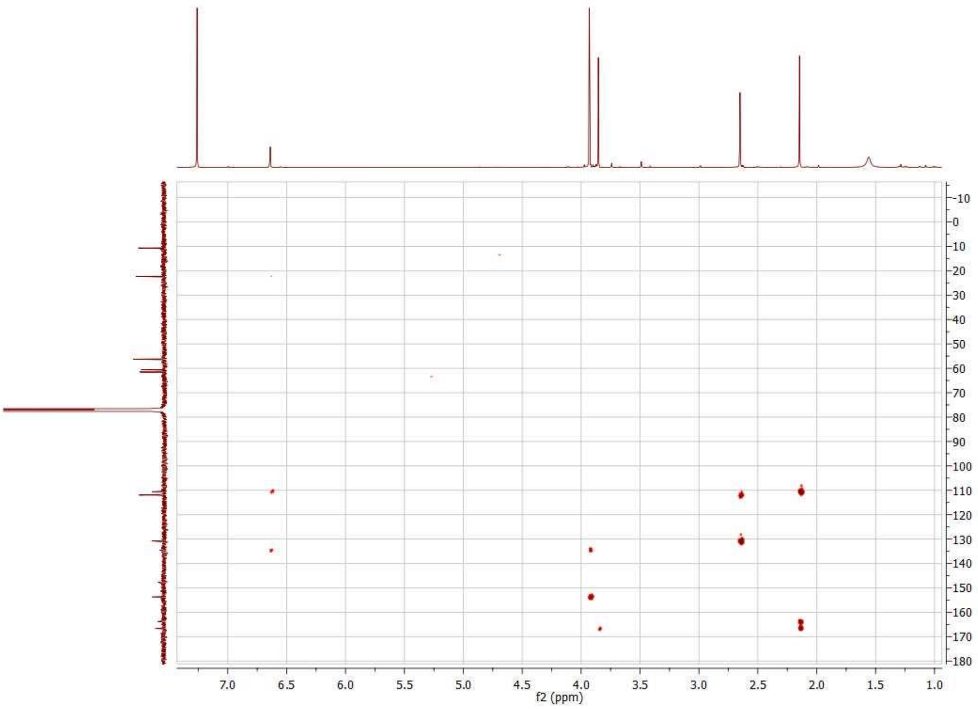

Figure S 6. HMBC spectrum of compound **1** ($CDCl_3$).

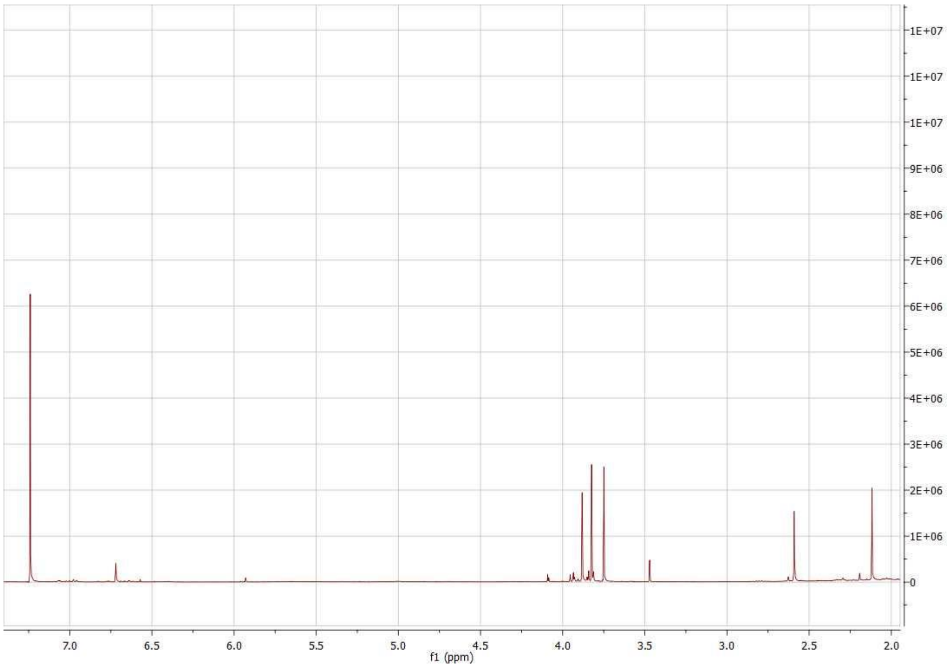

Figure S 7. ^{1}H-NMR spectrum of compound **2** ($CDCl_3$).

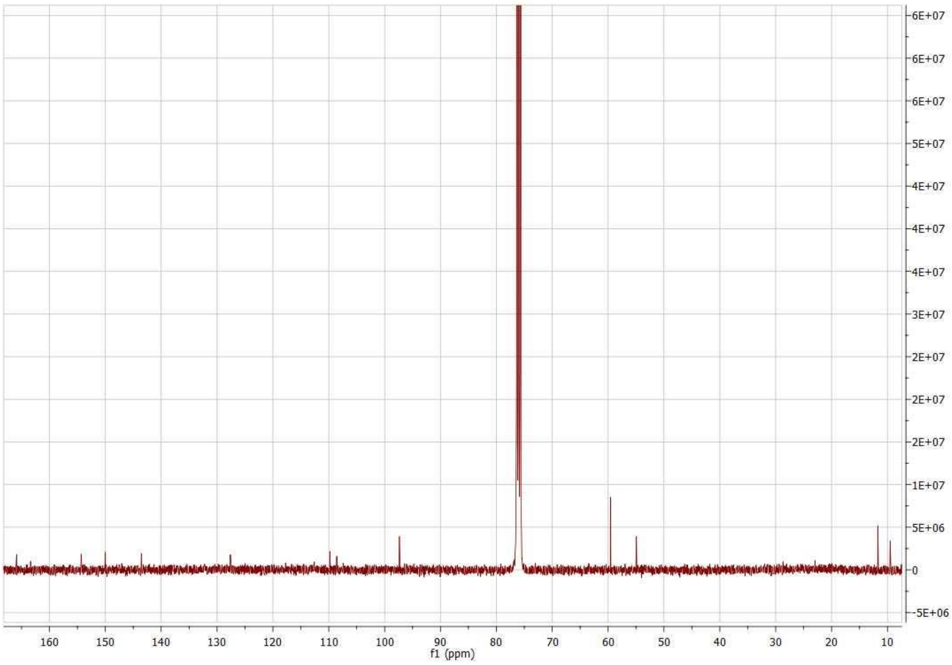

Figure S 8. ^{13}C-NMR spectrum of compound **2** ($CDCl_3$).

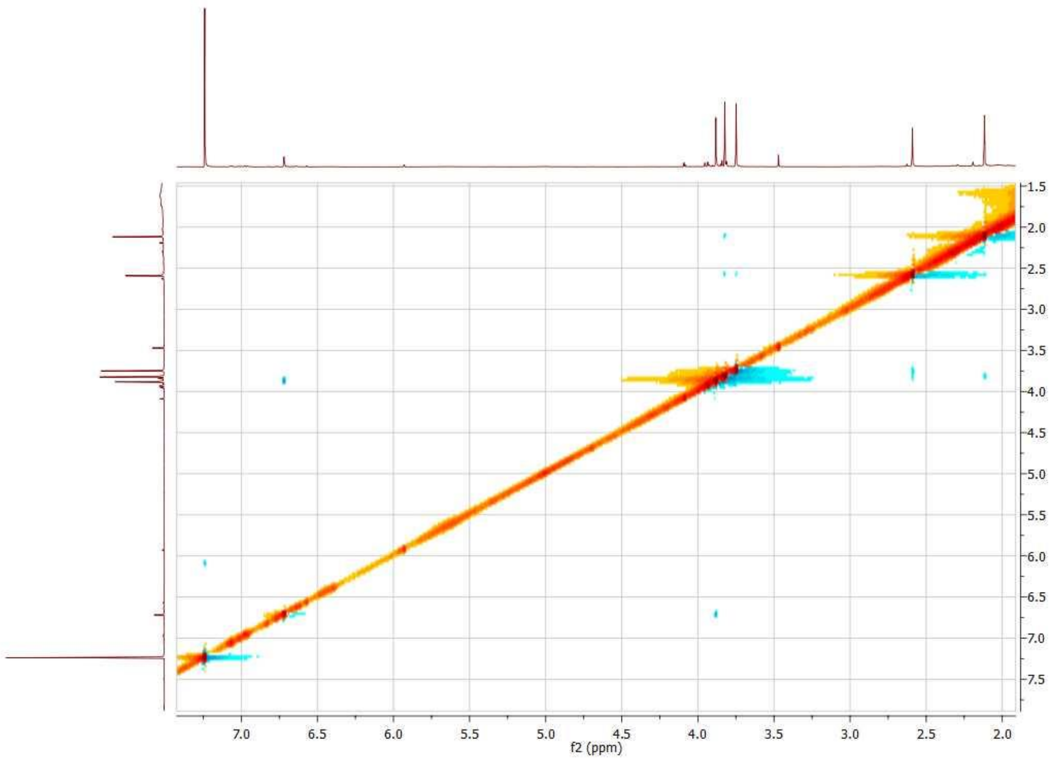

Figure S 9. NOESY spectrum of compound **2** ($CDCl_3$).

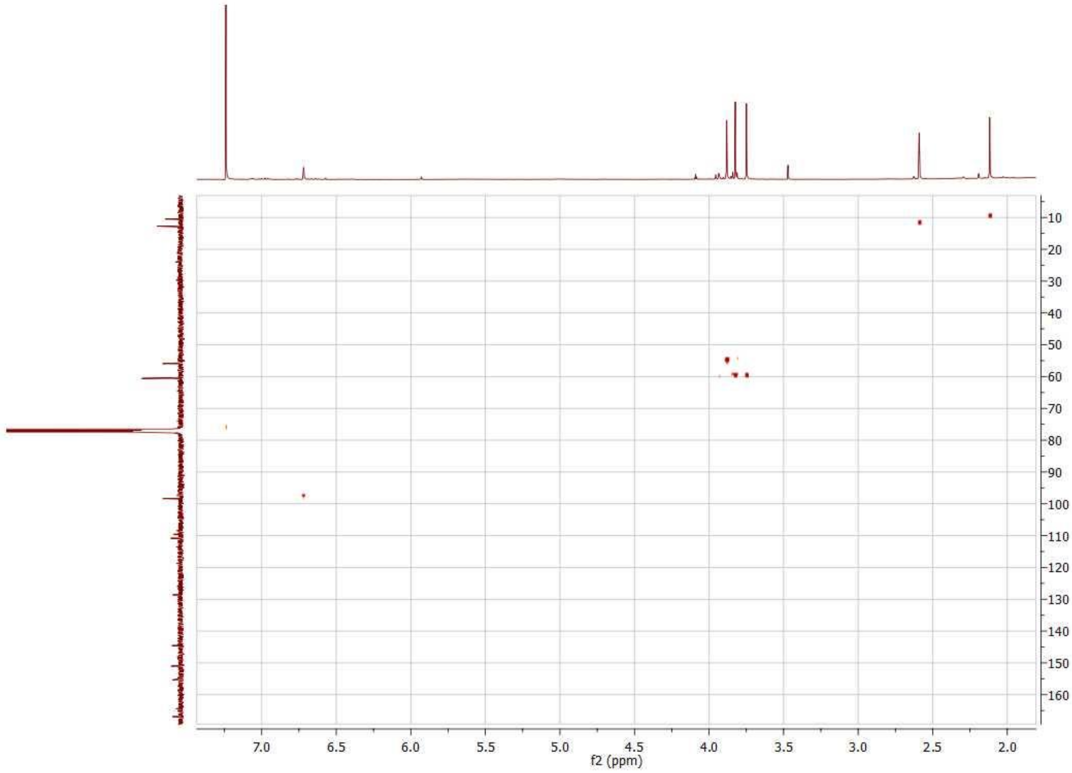

Figure S 10. HSQC spectrum of compound **2** ($CDCl_3$).

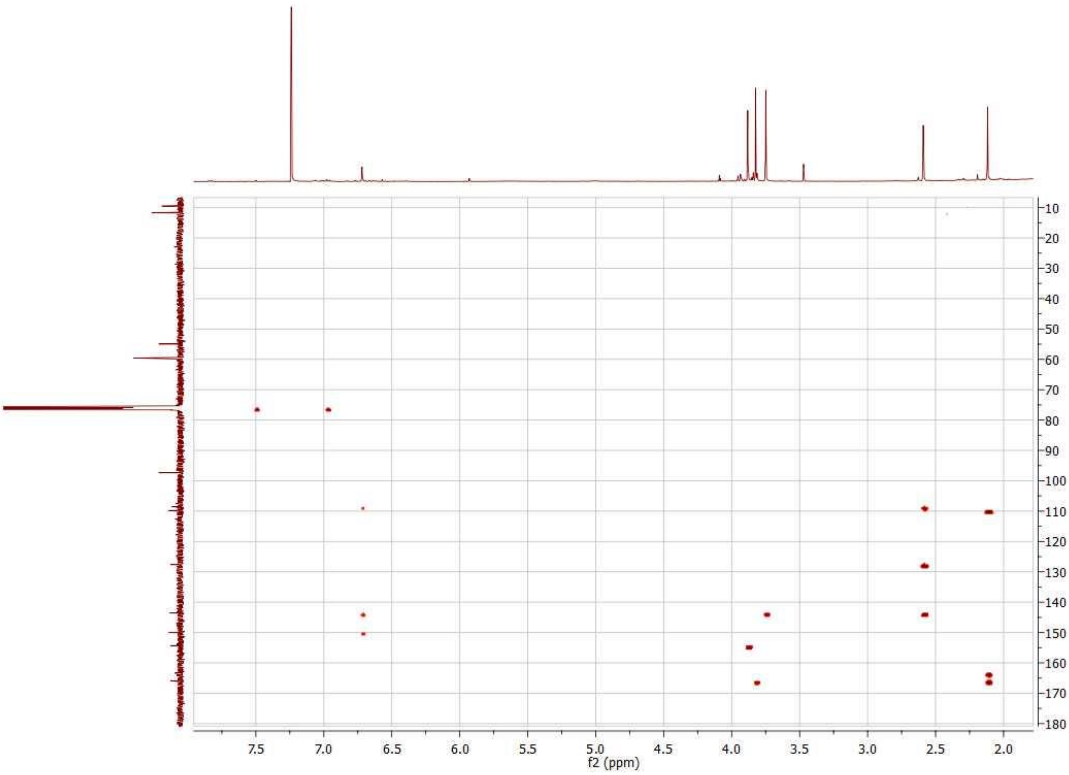

Figure S 11. HMBC spectrum of compound **2** ($CDCl_3$).
